# Preliminary landscape of *Candidatus* Saccharibacteria in the human microbiome

**DOI:** 10.3389/fcimb.2023.1195679

**Published:** 2023-07-27

**Authors:** Sabrina Naud, Camille Valles, Abdourahim Abdillah, Linda Abou Chacra, Fatima Zouina Mekhalif, Ahmad Ibrahim, Aurelia Caputo, Jean-Pierre Baudoin, Frédérique Gouriet, Fadi Bittar, Jean-Christophe Lagier, Stéphane Ranque, Florence Fenollar, Maryam Tidjani Alou, Didier Raoult

**Affiliations:** ^1^Aix-Marseille Université, IRD, APHM, MEPHI, IHU Méditerranée Infection, Marseille, France; ^2^Aix Marseille Université, IRD, AP-HM, SSA, VITROME, IHU Méditerranée Infection Marseille, France

**Keywords:** Candidate Phyla Radiation, *Candidatus* Saccharibacteria, human microbiome, electron microscopy, molecular detection

## Abstract

**Introduction:**

Candidate Phyla Radiation (CPR) and more specifically Candidatus Saccharibacteria (TM7) have now been established as ubiquitous members of the human oral microbiota. Additionally, CPR have been reported in the gastrointestinal and urogenital tracts. However, the exploration of new human niches has been limited to date.

**Methods:**

In this study, we performed a prospective and retrospective screening of TM7 in human samples using standard PCR, real-time PCR, scanning electron microscopy (SEM) and shotgun metagenomics.

**Results:**

Using Real-time PCR and standard PCR, oral samples presented the highest TM7 prevalence followed by fecal samples, breast milk samples, vaginal samples and urine samples. Surprisingly, TM7 were also detected in infectious samples, namely cardiac valves and blood cultures at a low prevalence (under 3%). Moreover, we observed CPR-like structures using SEM in all sample types except cardiac valves. The reconstruction of TM7 genomes in oral and fecal samples from shotgun metagenomics reads further confirmed their high prevalence in some samples.

**Conclusion:**

This study confirmed, through their detection in multiple human samples, that TM7 are human commensals that can also be found in clinical settings. Their detection in clinical samples warrants further studies to explore their role in a pathological setting.

## 1 Introduction

The evolution of culture-independent methods has been a defining factor in the study of the human microbiome. These technological advances have led to an unprecedented understanding of the human microbiome. Moreover, these studies have also highlighted a large proportion of unidentified sequences due to the significant number of uncultured microorganisms (Lok, 2015). The exploration of this so-called dark matter has led to the creation of additional divisions within the prokaryotic domains, namely Candidate Phyla Radiation (CPR) within the bacterial domain (Hugenholtz et al., 1998; Rinke et al., 2013) and Diapherotrites, Parvarchaeota, Aenigmarchaeota, Nanohaloarchaeota, and Nanoarchaeota (DPANN) within the archaeal domain (Rinke et al., 2013). In fact, Rheims et al. described the first sequence of the 16S rRNA gene of TM7 (later renamed *Candidatus* Saccharibacteria) in 1996 (Rheims et al., 1996). This was confirmed by subsequent phylogenetic studies showing the existence of 74 divisions or putative divisions, including the TM7 division (Hugenholtz et al., 1998; Castelle et al., 2018), and the reconstruction of genomes from metagenomes in the oral microbiota (Naud et al., 2022). CPR are widely spread in the environment and are found in aquatic environments such as freshwater lakes, seawater, groundwater as well as sediments, soils and plant rhizosphere (Ji et al., 2022). For instance, *Candidatus* Parcubacteria were detected in freshwater lakes (Linz et al., 2017) whereas *Ca. *Saccharibacteria and *Candidatus* Woesebacteria were found in ocean water (Tully et al., 2018). CPR are also widely spread in the human microbiome as they are found in several niches including the oral cavity, the gastrointestinal, urogenital, and respiratory tracts as well as the skin (Naud et al., 2022). Moreover, CPR are commensals of the oral microbiota with *Ca. *Saccharibacteria and *Ca. *Absconditabacteria as the most represented phyla. Interestingly, the phylum *Ca. *Saccharibacteria is the most represented in the human microbiome and has been detected in the gastrointestinal and urogenital tracts as well as the skin microbiota (Naud et al., 2022).

In this study, we specifically investigated the presence of *Ca. *Saccharibacteria in human samples through a retrospective and prospective study using molecular biology methods complemented with scanning electron microscopy (SEM). The objective was to describe the repertoire of *Ca. *Saccharibacteria in different human niches in physiological and clinical settings.

## 2 Materials and methods

### 2.1 Ethical approval

This study primarily used anonymized samples that were not specifically obtained for this context but rather were clinical samples remaining from diagnostic screenings. The patients were informed of the possible use of their samples for research purposes and retained their right to deny approval at any point. According to the French Jardé Law (Loi n° 2012–300 du 5 mars 2012 and Décret n° 2016–1537 du 16 Novembre 2016 published in the Journal Officiel de la République Française), as this noninvasive study did not involve the specific collection of samples or the use of medical/personal data from patients, neither institutional ethical approval nor individual patient consent was required. This general approach was validated by the ethical committee of the Méditerranée Infection Institute under agreement number n° 2019-002. The breast milk samples used in this study were collected as part of another study aiming to investigate the microbial diversity of these samples. This study was validated by the national ethical committee of Senegal under approval number SEN16/45 (Sarr et al., 2021). The participants gave informed and signed consent for this study. Both studies were conducted according to the guidelines of the Declaration of Helsinki.

### 2.2 Screening of clinical samples using molecular techniques

**Figure 1:**
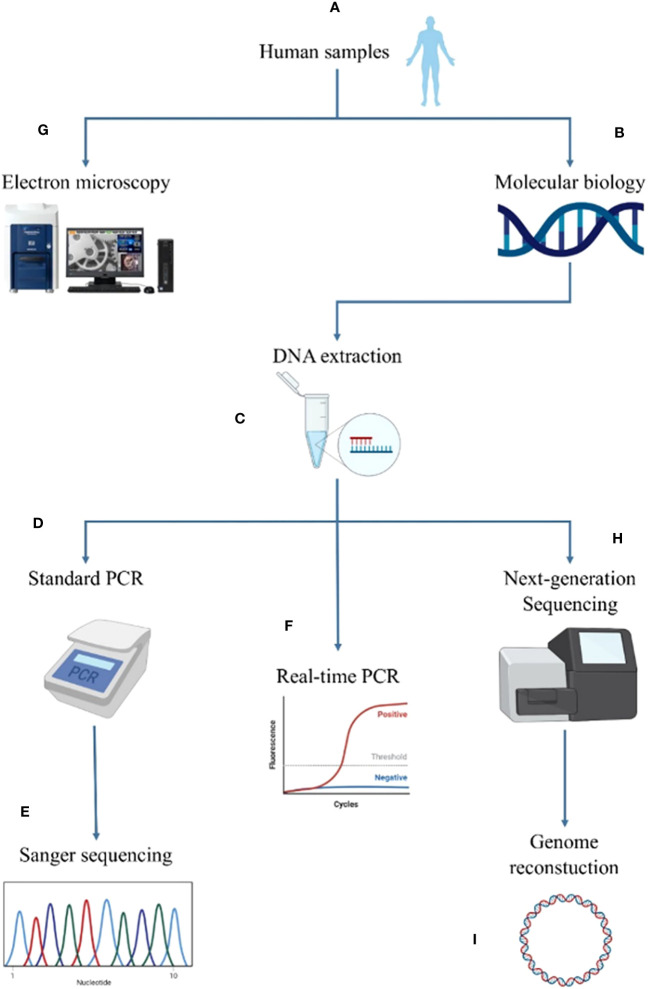
Summary of the screening methodology for *Candidatus* Saccharibacteria in the different human sites. The human samples **(A)** were first analyzed by molecular biological methods **(B)**. For this purpose, DNA was extracted **(C)** and analyzed using standard PCR **(D)** complemented by Sanger sequencing **(E)** and RT−PCR **(F)**. Imaging was performed for positive samples using tabletop electron microscopy **(G)**. Next-generation sequencing was performed for positive samples using molecular biology and electron microscopy **(H)** to reconstruct a new genome of *Candidatus* Saccharibacteria **(I)**.

*Ca. *Saccharibacteria are detected in samples using molecular methods, namely standard PCR (Sizova et al., 2015; Naud et al., 2022), real-time PCR (RT-PCR (Ibrahim et al., 2021; Naud et al., 2022)) as well as shotgun sequencing (Baker, 2022; Naud et al., 2022). Moreover, as *Ca. *Saccharibacteria cells have a small size and are uncultivable in axenic conditions, imaging methods such as electron microscopy and FISH are also used for their detection (Naud et al., 2022). In this study, each sample underwent two types of analyses, molecular biological and imaging (**Figure 1**). Molecular analyses included standard PCR, real-time PCR and shotgun sequencing whereas imaging was achieved through SEM.

#### 2.2.1 DNA extraction

##### 2.2.1.1 Biological fluids (oral, milk, urine, vaginal and blood samples)

DNA from clinical specimens was extracted using the EZ1 advanced XL biorobot (Qiagen, Courtaboeuf, France) with the EZ1 DNA tissue kit and the “DNA bacteria” extraction program. Two hundred microliters of each sample were used, and DNA elution was performed in a 100 µL volume.

##### 2.2.1.2 Solid specimens (fecal samples and cardiac valves)

The extraction of bacterial DNA from human fecal samples was performed using mechanical and chemical lysis as previously described (Guindo et al., 2020). For cardiac valve samples, DNA extraction was carried out as follows: first, we added 200 µL of buffer G2 (EZ1 DNA Tissue Kit) to the sample (10-40 mg) along with 10 µL of proteinase K (EZ1 DNA Tissue Kit). The sample was incubated at 56°C with an agitation of 350 rpm for 2 h. The digested sample was transferred to a new tube containing a tip of glass powder for mechanical lysis using Fastprep (MP Biomedicals, Instrument FastPrep-24 5G) for 40 seconds at maximum speed (6.5 m/s) before performing a ten minute-incubation at 100°C. After centrifugation (30 seconds at 13000 rpm), 200 µL of the supernatant was collected. Automated extraction using the EZ1 biorobot and the DNA Tissue kit (Qiagen) was then performed as described above with a final elution volume of 200 µL.

#### 2.2.2 TM7 molecular detection

TM7 molecular detection was achieved using standard PCR as well as real-time PCR (RT–PCR). Real-time PCR was performed using the primers and probe as previously described (**Table S1**) (Ibrahim et al., 2021). Briefly, 5 μL of DNA was mixed with 10 μL of MasterMix (Thermo Fisher, Illkirch-Graffenstaden, France), 0.5 μL of uracil DNA glycosylase (UDG), 3 μL of DNase/RNase-free ultrapure distilled water and 0.5 μL each of the forward primer, reverse primer and probe. The amplification and hybridization reactions were performed using a Light Cycler 480 with the Roche standard program. Standard PCR was performed using the M7580F and 1177R primer pair targeting the 16S ribosomal ribonucleic acid (rRNA) gene as previously described (Sizova et al., 2015). Sanger sequencing was also performed on positive samples as previously described (Drancourt et al., 2000) using the same primers used for standard PCR. The raw sequences were trimmed and assembled using ChromasPro software version 1.7 (Technelysium Pty Ltd., Tewantin, Australia). The obtained sequences were matched with the GenBank database using the BLASTN program (https://blast.ncbi.nlm.nih.gov/Blast.cgi, last accessed January 2023). The resulting sequences were then used to construct a phylogenetic tree using the software MEGA7 (Kumar et al., 2016) with the maximum likelihood method (Tamura 3 parameter model/method) and 500 bootstrap replicates. Alignment was conducted using Muscle v3.8.31 with default parameters.

#### 2.2.3 Next generation sequencing

Genomic DNA was sequenced with the paired-end strategy and was barcoded to be mixed with other genomic projects prepared with the Nextera XT DNA Sample Prep Kit (Illumina Inc., San Diego, CA, USA). To prepare the paired-end library, a dilution was performed to obtain 1 ng of each sample as input. The “tagmentation” step fragmented and tagged the DNA. Then, a limited cycle PCR amplification (18 cycles) completed the attachment of the tag adapters and introduced dual-index barcodes. After purification with AMPure XP beads (Beckman Coulter Inc, Fullerton, CA, USA), the libraries were then normalized using two different methods depending on the method of sequencing. For sequencing using a MiSeq sequencer (Illumina), libraries were normalized on specific beads according to the Nextera XT protocol (Illumina) and pooled together. Automated cluster generation and paired-end sequencing with dual index reads were performed in a single 39-hour run in 2x250 bp with a MiSeq reagent Kit (V2-500 cycles) (Illumina).

#### 2.2.4 Bioinformatic analysis

Reads were adjusted using Trimmomatic version 0.36.6 (Bolger et al., 2014) and assembled using the SPAdes software version 3.13.0 with default parameters (Bankevich et al., 2012). All contigs with a minimum length of 400 bp were conserved. BLASTn of these contigs versus nr was performed prior to the reconstruction of the genome into one single scaffold using the CONTIGuator tool (Galardini et al., 2011). Additionally, mapping of the obtained reads was performed using CLC Genomics Workbench (Liu and Di, 2020) against a reference genome of *Ca. *Saccharibacteria, *Ca*.

Nanosynbacter lyticus strain TM7x (GenBank accession number CP007496.1), using default settings with the length set at 0.9 and the similarity fraction set at 0.8. Moreover, all the reads obtained from 16S amplicon sequencing at the genomics platform of the University Hospital Méditerranée Infection Institute were analyzed using the MetaGX pipeline with the Silva 9.0 and culturomics databases (Diakite et al., 2019). MetaGX is an in-lab pipeline developed with the start-up XeGen (http://xegen.eu/) based on QIIME (Caporaso et al., 2010) using BLAST (Altschul et al., 1990) for taxonomic assignment and SILVA (Quast et al., 2013; Yilmaz et al., 2014) as a reference dictionary. For taxonomic assignments, we only included OTUs consisting of at least 20 reads. The OTUs were then searched against each database using BLASTN. The best match (≥ 97% identity and 100% coverage) was retrieved for each OTU from the reference database, and taxonomy was assigned up to the species level.

The database consisted of 3,788 samples at the time our analysis was conducted. Nonhuman samples (including animal, environmental, insect and unidentified samples) were excluded from this study. A total of 3,550 human samples were ultimately included in our analysis.

### 2.3 Sample screening using scanning electron microscopy

All samples except cardiac valves were fixed in a 2.5% glutaraldehyde fixative solution for at least one hour. Using the cytospin instrument, the sample was spotted on the slide and contrasted using a 1% aqueous phosphotungstic acid (PTA) solution (pH 7.0) for two minutes. The slide was then air-dried and observed using electron microscopy as previously described. Electron micrographs were acquired using a Hitachi TM4000 Plus benchtop scanning electron microscope (Hitachi, Tokyo, Japan). Cardiac valve samples were prepared with a one-hour fixation with 2.5% glutaraldehyde solution before cutting with a razor blade. The samples were rinsed twice, first with 0.1 M Caco and then with distilled water for 1 minute each. Dehydration of the cardiac valves was performed under agitation in five successive two-minute ethanol baths (20%, 50%, 70%, 90%, and 100%). We then performed two 5-min successive incubations of the valve sample, first in a bath consisting of 100% ethanol (1 V) and 100% hexamethyldisilazane (HMDS) (2 V), followed by a second bath consisting of 100% HMDS solely. The valve was dried under a laminar flow hood for 1 h and a cross-section was deposited on a glass slide. The observation was performed using a Hitachi SU5000 scanning electron microscope (Hitachi, Tokyo, Japan). In this study, we considered *Ca. *Saccharibacteria as a bacterial symbiont or free structure with widths ranging from 100 nm to 500 nm, as previously described (He et al., 2015; Cross et al., 2019; Ibrahim et al., 2021). This identification was presumptive and based on the current state of knowledge on CPR. CPR were identified based on SEM micrograph of a coculture of *Schaalia odontolytica* with *Ca. *Saccharibacteria (**Figure S1**) were obtained in a separate study by our team.

### 2.4 Statistical analysis

Statistical analyses were performed using R (Chan, 2018), GraphPad Prism 8 and OpenEpi (https://www.openepi.com/Menu/OE_Menu.htm). Normality and variances were determined using R (Shapiro test and Bartlett test, respectively). Quantitative comparisons were conducted using the Mann−Whitney test for two variables and the Kruskal−Wallis test for three variables or more.

## 3 Results

### 3.1 Prevalence of CPR in human samples from the MetaGX database

The 16S amplicon datasets from the MetaGX database were analyzed to assess the prevalence and abundance of CPR within the human microbiome (Diakite et al., 2019). Seven CPR phyla, namely, *Ca. *Saccharibacteria, *Ca. *Atribacteria, *Ca. *Parcubacteria, *Ca. *Microgenomates, *Ca. *Dojkabacteria, *Ca. *Gracilibacteria and *Ca. *Katanobacteria were detected in these datasets (**Table 1**). *Ca*.

**Table 1:** Relative abundance of CPR phyla in different human niches.

Samples	*Candidatus* Saccharibacteria	*Candidatus* Atribacteria	*Candidatus* Parcubacteria	*Candidatus* Microgenomates	*Candidatus* Dojkabacteria	*Candidatus* Gracilibacteria	*Candidatus* Katanobacteria
Oral cavity	2.92E-02	0.00E+00	0.00E+00	0.00E+00	0.00E+00	0.00E+00	0.00E+00
Sputum	2.85E-02	0.00E+00	0.00E+00	0.00E+00	0.00E+00	0.00E+00	0.00E+00
Respiratory tract	4.98E-03	7.24E-06	1.10E-05	7.08E-06	2.55E-06	1.58E-05	0.00E+00
Gastro-intestinal tract	3.84E-03	1.30E-05	0.00E+00	0.00E+00	0.00E+00	0.00E+00	0.00E+00
Urinary tract	3.64E-03	9.19E-05	1.47E-04	1.12E-04	2.70E-05	0.00E+00	5.61E-05
Human breast milk	2.70E-03	1.25E-04	8.42E-06	0.00E+00	0.00E+00	2.48E-06	0.00E+00

This table is presented as a heatmap.

Saccharibacteria was the most represented phylum in all the analyzed sample types (oral cavity, sputum, gastrointestinal tract, respiratory tract, urinary tract, and human breast milk). A total of 28.9% (1,026/3,550) of the samples presented reads assigned to the CPR and the phylum *Ca. *Saccharibacteria was the most represented. One hundred percent (9/9) of the sputum samples, 94.7% (18/19) of the oral samples, 37% (210/568) of the respiratory samples, 35.4% (124/350) of the breast milk samples, 29.5% (538/1,826) of fecal samples and 17.6% (121/686) of the urine samples presented OTUs assigned to *Ca. *Saccharibacteria (**Table 2**). A few reads assigned to *Ca*.

**Table 2:** Summary table of the methodologies used to screen CPR in this study.

Samples	Standard PCR	Sanger Sequencing	RT−PCR	Microscopy	Mapping (Total metagenomic)	16S amplicon datasets	Positive correlation
Oral cavity	89.2% (58/65)		96.9% (95/98)		Proof of concept	94.7% (18/19)	
Gastrointestinal tract	65.8% (50/76)		52.6% (40/76)		Proof of concept	29.5% (538/1826)	
Urine	8.8% (14/160)		0.6% (1/160)			17.6% (121/686)	
Human breast milk	2.7% (5/182)		21.4% (39/182)			35.4% (124/350)	
Respiratory tract						37% (210/568)	
Sputum						100% (9/9)	
Vagina	3.1% (9/288)		6.6% (19/288)			0% (0/1)	
Blood	0.8% (63/7405)					0% (0/20)	
Cardiac valves	2.5% (17/670)		1.2% (8/693)				
Skin						15.4% (4/26)	
Bones						2% (1/50)	
Male genital organs						3.8% (1/26)	
Cerebral abscesses						0% (0/1)	

Green, methodology used with positive results; gray, methodology not used; and red, methodology used with negative results.

**Figure 2:**
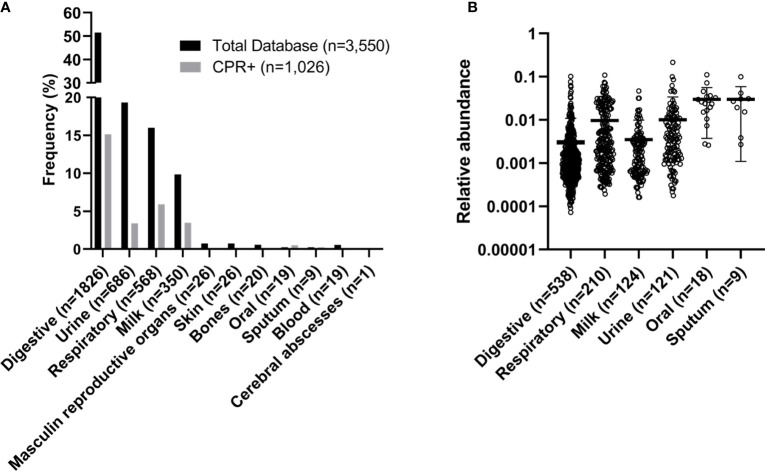
Frequency and relative abundance of samples with OTUs assigned to CPR. **(A)** Frequency and **(B)** relative abundance within the human microbiome.

Saccharibacteria were found in skin, bone, and male genitalia samples (15.4% (4/26), 2% (1/50), 3.8% (1/26) samples, respectively). In addition, no reads of *Ca. *Saccharibacteria were found in human blood and cerebral abscess samples (**Figure 2A**). The oral microbiota (oral and sputum samples) exhibited a higher prevalence of CPR than the other studied niches (p-value<0.0001, Kruskal-Wallis test) (**Figure 2B**, **Table S2**).

### 3.2 Retrospective screening of *Candidatus* Saccharibacteria in clinical samples

**Figure 3:**
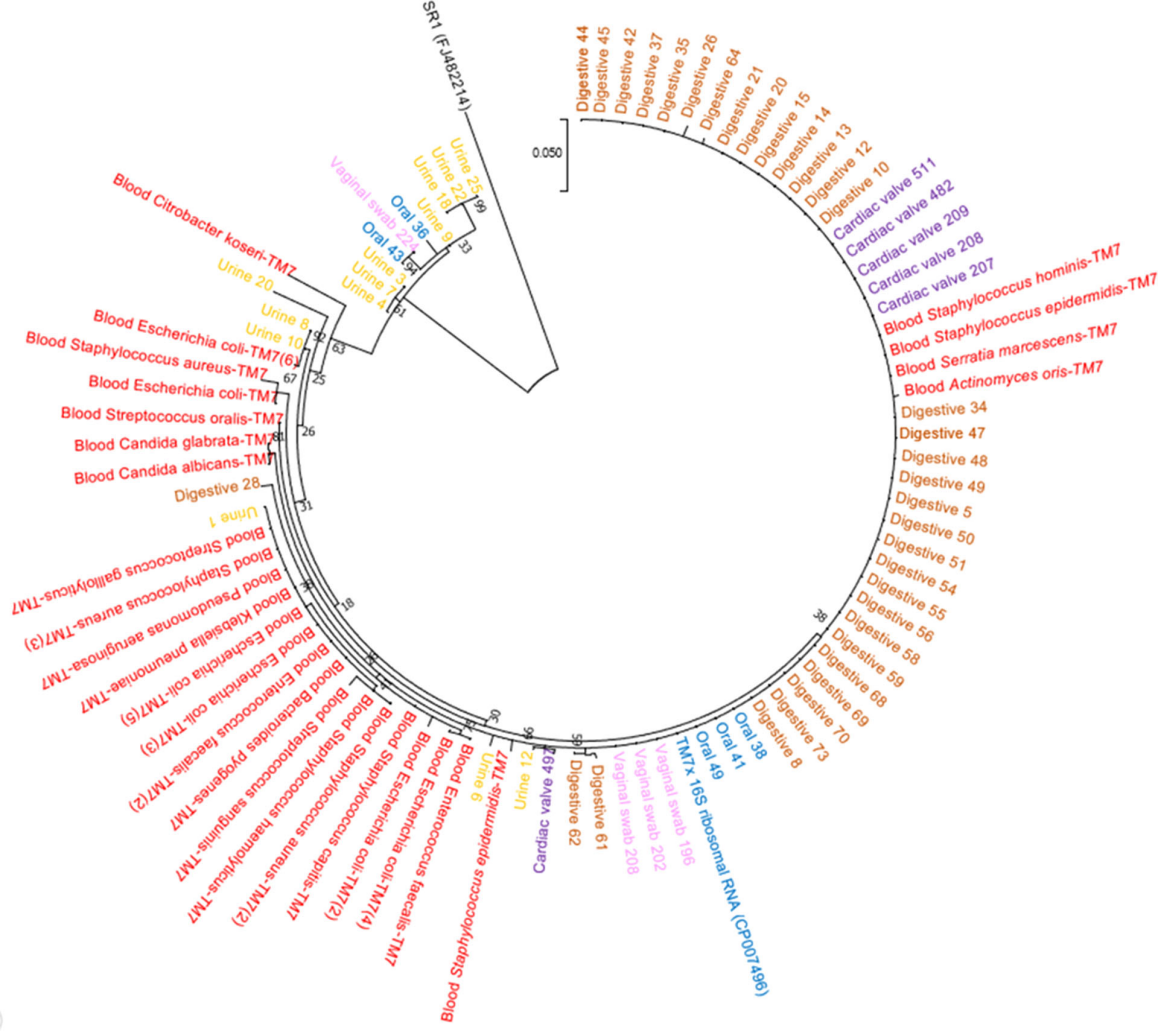
Phylogenetic tree based on the amplicon sequences constructed using MEGA7. Red, amplicons from blood cultures; yellow, amplicons from urine samples; brown, amplicons from fecal samples; pink, amplicons from vaginal samples; purple, amplicons from cardiac valves; and blue, amplicons from oral samples.

After analysis of our 16S amplicon database, we performed a retrospective study and screened for the presence of *Ca. *Saccharibacteria in different types of clinical samples. The screening was performed using standard PCR and real-time PCR for the initial assessment, which was then confirmed by observation of *Ca. *Saccharibacteria using scanning electron microscopy and Sanger sequencing (**Figure 3**, **Table S3**) of the PCR products of positive samples (**Table 2**). Finally, we were able to reconstruct two CPR bacterial genomes from the mapping of reads obtained from shotgun metagenomics of stool and oral cavity samples (**Table 2**).

#### 3.2.1 Human oral samples

**Figure 4:**
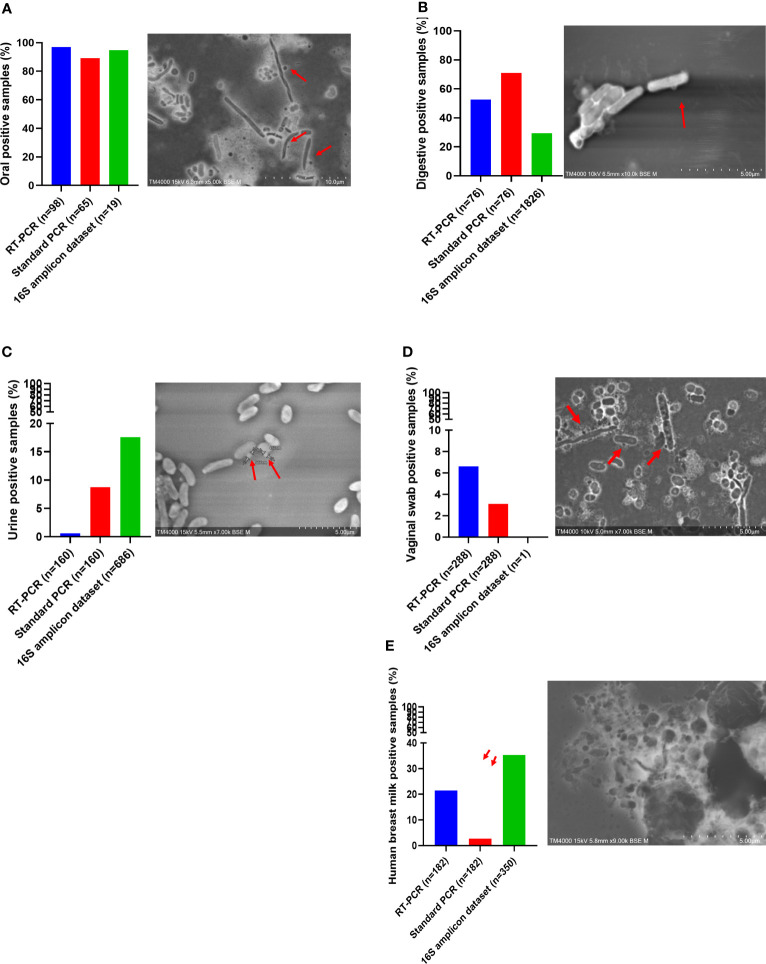
Screening of *Candidatus* Saccharibacteria using molecular biology and electron microscopy in anatomical sites. *Candidatus* Saccharibacteria were detected in oral samples **(A)**, fecal samples **(B)**, human breast milk samples **(C)**, urine samples **(D)**, and vaginal swab samples **(E)**.

Oral samples (dental plaque) exhibited the highest prevalence of *Ca. *Saccharibacteria. They were detected in 97% (95/98) of positive samples using RT–PCR and 89.2% (58/65) of positive samples using standard PCR (**Figure 4A**). Ct values ranged from 16.93 to 31.90 with an average Ct of 23.41 (**Figure 4A**). Additionally, Sanger sequencing of the PCR amplicons of approximately 600 bp showed homology with the 16S rRNA of *Ca. *Saccharibacteria (**Figure 3**, **Table S3**). Structures compatible with those of CPR (cocci-like shapes, free or associated with bacteria with a diameter under 500 nm) according to the literature (He et al., 2015; Bor et al., 2016; Ibrahim et al., 2021; Naud et al., 2022) were observed in human oral specimens (**Figure 4A**) using SEM in positive samples. The mapping-based assembly of reads obtained from the shotgun sequencing of a positive oral sample (Ct=19.04) allowed the reconstruction of a genome with 81% coverage of the reference genome (GenBank accession number CP007496.1). This genomic sequence consisted of one scaffold with a size of 0.7 Mbp and a GC% of 43.42. The 16S rRNA gene (GenBank accession number OX335640) shared a similarity of 100% with that of *Ca. *Nanosynbacter sp. HMT-352 strain TM7-037 (CP089288.1), whereas the maximum digital DNA−DNA hybridization (dDDH) (Auch et al., 2010; Meier-Kolthoff et al., 2013) of 70.7% was shared with *Ca. *Minimicrobia massiliensis (GenBank accession number CADDWL010000000).

#### 3.2.2 Human fecal samples

Fecal samples also presented a prevalence of *Ca. *Saccharibacteria and accounted for 52.6% (40/76) of the positive samples based on the RT–PCR results, with a mean Ct of 28.55 [24.53-33.33] (**Figure 4B**). Additionally, 65.8% (50/76) of fecal samples were positive for *Ca. *Saccharibacteria using standard PCR (**Figure 4B**), and the corresponding sequences matched *Ca. *Saccharibacteria (**Figure 3**, **Table S3**). This was confirmed with the SEM micrographs showing structures consistent with these minimicrobes in positive fecal samples (**Figure 4B**). Moreover, two genomes (GenBank accession numbers CADDWL010000000 and CAJGBL010000000) were reconstructed from the mapping of reads generated using shotgun sequencing of two fecal samples (Ct 24.65 and Ct 27.55) against *Ca. *Nanosynbacter lyticus TM7x (GenBank accession number CP007496.1). These genomes consisted of 91 and 197 contigs, resulting in a size of 0.9 Mbp and 0.7 Mbp with a 43.2% and 43.7% GC content, respectively. These two genomic sequences shared a dDDH value of 56.8 [54.0-59.5].

#### 3.2.3 Urogenital microbiota

*Ca.* Saccharibacteria were also detected in the urogenital tract. Urine samples showed a prevalence of 0.6% (1/160, **Figure 4C**) using RT–PCR and 8.8% (14/160) using standard PCR. A higher prevalence was obtained in vaginal samples using RT–PCR (6.6% (19/288)), with Ct values ranging from 22.35 to 34.88 (**Figure 4D**), and 3.1% (9/288) for standard PCR. These results were confirmed with Sanger sequencing of the PCR products (**Figure 3**, **Table S3**) and SEM observation of structures consistent with those of CPR in positive samples (**Figures 4C, D**).

#### 3.2.4 Human breast milk

Human breast milk was an unexpected niche for CPR. These samples showed a non-negligible prevalence for CPR with 21.4% (39/182) of breast milk samples positive using RT–PCR (33.00 [28.94; 34.92]) (**Figure 4E**), whereas a lower prevalence was obtained using standard PCR, with 2.7% (5/182) of positive breast milk samples (**Figure 4E**). Moreover, symbiotic structures were observed in positive breast milk samples using SEM (**Figure 4E**).

#### 3.2.5 Human clinical samples (blood and cardiac valves)

**Figure 5:**
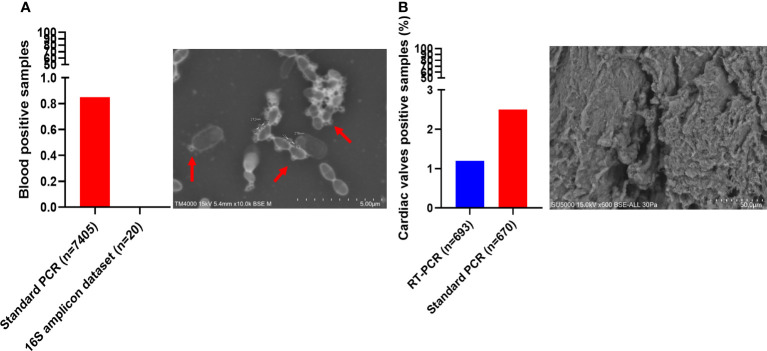
Screening of *Candidatus* Saccharibacteria using molecular biology and electron microscopy in clinical samples. *Candidatus* Saccharibacteria were detected in blood cultures **(A)** and cardiac valve samples **(B)**.

Interestingly, *Ca. *Saccharibacteria were also detected in clinical samples at a low frequency. A large screening of 7,405 positive blood culture bottles from febrile patients showed that 0.8% (63/7,405) were positive using standard PCR targeting *Ca. *Saccharibacteria (**Figure 5A**). These data were confirmed by Sanger sequencing of the PCR products (**Figure 3**, **Table S3**). The most common bacterial species associated with *Ca. *Saccharibacteria were *Escherichia coli* (15%), *Klebsiella pneumoniae* (11%), *Staphylococcus aureus* (11%), *Staphylococcus epidermidis* (10%), *Staphylococcus capitis* (10%), and *Enterococcus faecalis* (6%). Strikingly, we also detected *Ca. *Saccharibacteria in the blood culture of a patient infected by the yeast *Candida albicans* (3%). These results were confirmed using SEM that revealed structures consistent with CPR in positive blood cultures (**Figure 5A**). Cardiac valve samples showed a prevalence of 1.2% (8/693) using RT–PCR, including 75% (6/8) of control valves (valves not affected by infectious endocarditis) and 25% (2/8) of valves from patients with infective endocarditis caused by *Serratia marcescens* and *Streptococcus mitis* (**Figure 5B**). Standard PCR screening detected *Ca. *Saccharibacteria in 2.5% (17/670) of cardiac valve samples (**Figure 5B**). However, their presence could not be confirmed using SEM (**Figure 5B**).

## 4 Discussion

Until recently, CPR were overlooked within the bacterial domain and therefore in the human microbiome. Their recent discovery has led to their increased detection in 16S amplicon sequencing datasets as well as those of metagenomics, thus highlighting their non-negligible presence in the oral cavity. Here, we have carried out a preliminary study exploring the prevalence of CPR, and more specifically *Ca. *Saccharibacteria in various human sample types through a retrospective analysis of CPR within our in-lab database of 3,550 16S amplicon datasets as well as prospective analysis of readily available human samples. Thus, we highlighted the ubiquitous presence of CPR, specifically that of *Ca. *Saccharibacteria, in the human microbiome. We demonstrated the quasi-ubiquitous presence of *Ca. *Saccharibacteria in the oral cavity as previously described (Bor et al., 2016; Bor et al., 2019, Cross et al., 2019; Bor et al., 2020; Naud et al., 2022). *Ca. *Saccharibacteria were also detected in other human niches, including the gastrointestinal tract, urogenital tract, and breast milk, as well as in clinical samples, including blood samples and heart valves. The detection of CPR through molecular methods was confirmed by visualization via electron microscopy, which revealed structures under 500 nm generally attached to bacterial hosts. It is noteworthy that better detection rates were achieved using RT–PCR targeting 23S rRNA than using standard PCR targeting the 16S rRNA gene. The RT−PCR system presented a high specificity due to its design based on all complete *Ca. *Saccharibacteria genomes available at the time of the design (Ibrahim et al., 2021), in contrast to the standard PCR system, which presented a higher sensitivity as it was designed based on the 16S rRNA gene (Takenaka et al., 2018). Despite the discrepancy between the two methods, both highlighted the oral microbiota as exhibiting the highest prevalence of *Ca. *Saccharibacteria as described in the literature (Heller et al., 2016; Oliveira et al., 2016; Bor et al., 2020; Murugkar et al., 2020), followed by the gastrointestinal tract, breast milk, vaginal tract and urine. To our knowledge, this study represents the largest screening of CPR in humans. We provide a more complete picture of the repartition of *Ca. *Saccharibacteria within the human microbiome through the addition of breast milk as a previously unknown niche. Moreover, *Ca. *Saccharibacteria were also detected in pathogenic settings, specifically in the blood of febrile patients and cardiac valves of patients suffering from infectious endocarditis. This is the first reported detection in cardiac valves whereas *Ca. *Parcubacteria, another CPR phylum, has previously been reported within human blood (Kowarsky et al., 2017; Chen et al., 2020). This study warrants further exploration of CPR prevalence and relative abundance, including phyla other than *Ca. *Saccharibacteria, in larger sample sizes and additional niches such as the skin and respiratory tract, in which CPR have been previously detected (Zhou et al., 2013; McLean et al., 2020; Rueca et al., 2021). Furthermore, case−control studies could be conducted to specifically assess the impact of CPR on dysbiosis.

## Data availability statement

The datasets presented in this study can be found in online repositories. The names of the repository/repositories and accession number(s) can be found in the article/**Supplementary Material**.

## Ethics statement

The studies involving human participants were reviewed and approved by the Ethical committee of the Méditerranée Infection Institute and the National ethical committee of Sénégal. Written informed consent was not provided because this study primarily used anonymized samples that were not specifically obtained for this context but rather were clinical samples remaining from diagnostic screenings. The patients were informed of the possible use of their samples for research purposes and retained their right to deny approval at any point. According to the French Jardé Law (Loi n° 2012–300 du 5 mars 2012 and Décret n° 2016–1537 du 16 Novembre 2016 published in the Journal Officiel de la République Française), as this noninvasive study did not involve the specific collection of samples or the use of medical/personal data from patients, neither institutional ethical approval nor individual patient consent was required. This general approach was validated by the ethical committee of the Méditerranée Infection Institute under agreement number no 2019-002. The breast milk samples used in this study were collected as part of another study aiming to investigate the microbial diversity of these samples. This study was validated by the national ethical committee of Senegal under approval number SEN16/45 (Sarr et al., 2021). The participants gave informed and signed consent for this study. Both studies were conducted according to the guidelines of the Declaration of Helsinki.

## Author contributions

Conceptualization, MT and DR; Data curation, AC; Formal analysis, SN, CV, AA, LA, FM and AI; Funding acquisition, DR; Investigation, SN, CV, AA, LA, FM, AI and J-PB; Methodology, MT and DR; Resources, FG, FB and FF; Supervision, J-CL, SR, MT and DR; Validation, J-CL, SR, FF, MT and DR; Visualization, SN and CV; Writing – original draft, SN, CV, MT and DR; Writing – review & editing, MT and DR.
