# Supplementary material for: Preliminary landscape of Candidatus Saccharibacteria in the human microbiome
Source: Front Cell Infect Microbiol. 2023 Jul 27;13:1195679. doi: 10.3389/fcimb.2023.1195679 (PMC10414567; doi:10.3389/fcimb.2023.1195679)
Supplement: Supplementary file 1 [file DataSheet_1.docx]

**Preliminary landscape of *Candidatus* Saccharibacteria in the human microbiome**

Sabrina Naud^1#^, Camille Valles^1#^, Abdourahim Abdillah^2^, Linda Abou Chacra^2^, Fatima Zouina Mekhalif ^1^, Ahmad Ibrahim^1^, Aurelia Caputo^1^, Jean-Pierre Baudoin^1^, Frédérique Gouriet^1^, Fadi Bittar^1^, Jean-Christophe Lagier, Stéphane Ranque^2^, Florence Fenollar^2^, Maryam Tidjani Alou^1^ and Didier Raoult^1*^

^#^Equally contributing co-authors

^1^Aix Marseille Univ, IRD, AP-HM, MEPHI, IHU-Méditerranée Infection, Marseille, France

^2^Aix Marseille Univ, IRD, AP-HM, SSA, VITROME, IHU-Méditerranée Infection Marseille, France

^*^Corresponding author: Prof. Didier Raoult, Institut Hospitalo-Universitaire Méditerranée Infection, 19-21 Boulevard Jean Moulin 13385 Marseille Cedex 05, France. Phone: + 33 (0) 4 13 73 24 01. Fax: + 33 (0) 4 13 73 24 02. [didier.raoult@gmail.com](mailto:didier.raoult@gmail.com)


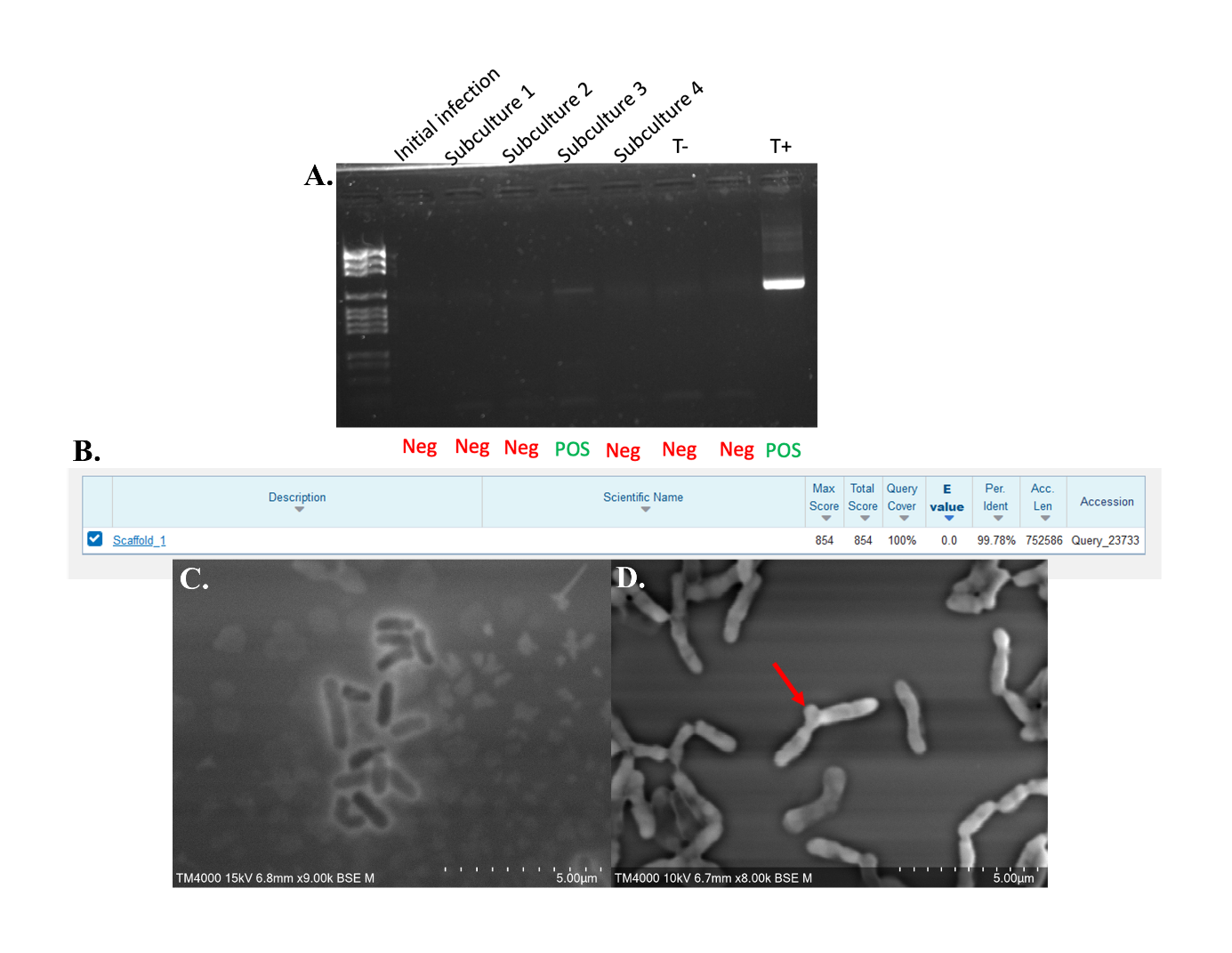


Figure S1: **Coculture image of *Candidatus* Saccharibacteria associated with its host *Schaalia odontolytica.*** A fecal sample positive to *Candidatus* Saccharibacteria was filtered at 0.22µm, then ultracentifuged as previously described ^24^. The filtrate was cultured with *S. odontolytica* then subcultured 4 times every 24 hours. We realized standard PCR of these cultures (A) and then sequenced the PCR products by Sanger sequencing. Blast results confirm the detection of *Candidatus* Saccharibacteria in these cultures and showed 100% coverage and 99.78% identity with *Candidatus* Saccharibacteria (B). Exosymbionts were observed using scanning electron microscopy. (C) An uninfected *S. odontolytica* culture. (D) Infected *S. odontolytica* showing deformed bacilli with an exosymbiont. These micrographs were used as a positive control in this study.

**Table S1. Summary table of primers and probes used to perform standard PCR and RT–PCR, respectively.**

| PCR types | Primer and probe names | Sequences | References |
| --- | --- | --- | --- |
| Standard PCR | 1177R | GACCTGACATCATCCCCTCCTTCC | Brinig et al., 2003 |
| Standard PCR | 580F | AYTGGGCGTAAAGAGTTGC | Hugenholtz et al., 2001 |
| RT–PCR | SacchariF | GGCTTATAGCGCCCAATAG | Ibrahim et al., 2021 |
| RT–PCR | SacchariR | CGGATATAAACCGAACTGTC | Ibrahim et al., 2021 |
| RT–PCR | SacchariP | 6-FAM-CATAGACGGCGCTGTTTGGCAC-TAMRA | Ibrahim et al., 2021 |

**Table S2. Kruskal-Wallis statistical tests of the relative abundance of CPR-positive samples using 16S amplicon sequencing.**

**Table S3. Summary table of nucleotide sequences cleaned and assembled using ChromasPro obtained using Sanger sequencing.**

Sequences were compared with the NCBI NR database using the BLASTn program (<https://blast.ncbi.nlm.nih.gov/Blast.cgi>).
